# Co-introduction of precipitate hardening and TRIP in a TWIP high-entropy alloy using friction stir alloying

**DOI:** 10.1038/s41598-021-81350-0

**Published:** 2021-01-15

**Authors:** Tianhao Wang, Shivakant Shukla, Bharat Gwalani, Subhasis Sinha, Saket Thapliyal, Michael Frank, Rajiv S. Mishra

**Affiliations:** 1 Department of Materials Science and Engineering, University of North Texas, Denton, TX 76207 USA; 2 Present Address: Pacific Northwest National Laboratory, Richland, WA 99352 USA; 3 Present Address: Oak Ridge National Laboratory, Oak Ridge, TN 37831 USA; 4 Present Address: Department of Metallurgical Engineering, IIT(BHU), Varanasi, India

**Keywords:** Mechanical properties, Metals and alloys

## Abstract

Tuning deformation mechanisms is imperative to overcome the well-known strength-ductility paradigm. Twinning-induced plasticity (TWIP), transformation-induced plasticity (TRIP) and precipitate hardening have been investigated separately and have been altered to achieve exceptional strength or ductility in several alloy systems. In this study, we use a novel solid-state alloying method—friction stir alloying (FSA)—to tune the microstructure, and a composition of a TWIP high-entropy alloy by adding Ti, and thus activating site-specific deformation mechanisms that occur concomitantly in a single alloy. During the FSA process, grains of the as-cast face-centered cubic matrix were refined by high-temperature severe plastic deformation and, subsequently, a new alloy composition was obtained by dissolving Ti into the matrix. After annealing the FSA specimen at 900 °C, hard Ni–Ti rich precipitates formed to strengthen the alloy. An additional result was a Ni-depleted region in the vicinity of newly-formed precipitates. The reduction in Ni locally reduced the stacking fault energy, thus inducing TRIP-based deformation while the remaining matrix still deformed as a result of TWIP. Our current approach presents a novel microstructural architecture to design alloys, an approach that combines and optimizes local compositions such that multiple deformation mechanisms can be activated to enhance engineering properties.

## Introduction

High-entropy alloys (HEAs) have attracted considerable interest since they were first conceptualized and synthesized by Yeh^1^. This ongoing interest is due mainly to HEAs maintaining excessive entropy-driven phase stability and unique mechanical properties that include high strength at both room temperature and high temperature, good wear resistance, corrosion resistance and fatigue properties^2,3^. HEAs also provide a completely new compositional space for designing novel materials with exceptional properties. Recent developments in the field of non-equiatomic HEAs have focused on introducing TRIP (transformation-induced plasticity)^4^, TWIP (twinning-induced plasticity)^5^, and joint activation of TWIP and TRIP^6,7^ by designing metastable alloys. Moreover, researchers have also incorporated precipitation hardening (PH)^8^ and interstitial solid solution^7^ in the HEAs as additional strengthening mechanisms. The preliminary study on TRIP-DP (dual phase)-HEA (Fe_50_Mn_30_Co_10_Cr_10_ at.%, γ (*FCC*) and ε (*HCP*) phases) by Li et al.^4^ demonstrated that both strength and ductility can be improved by a strain-induced γ to ε transformation. Inspired by this finding, Nene et al.^9^ modified the alloy composition by adding Si and increasing Cr (Fe_42_Mn_28_Co_10_Cr_15_Si_5_ at.%) and thus, increasing metastability of the γ phase to capitalize on the TRIP effect. Meanwhile, Sinha et al.^5^ exchanged Co for Ni (Fe_42.8_Mn_28.3_Cr_14.8_Ni_9.6_Si_4.5_ at.%) to increase the stacking fault energy to favor TWIP during plastic deformation^10–12^. Hence, the previous work demonstrated the possibility of both TWIP and TRIP in this alloy system through compositional tuning.

Furthermore, a combined body of literature^5–9^ suggests that even minor alloying addition can greatly impact HEA strengthening mechanisms such as TRIP, TWIP and PH. Yet, the limited development of alloys can be attributed to conventional development methods that include casting, thermomechanical processing, and annealing process. In this study, friction stir alloying (FSA), a novel solid-state alloying method derived from friction stir processing (FSP)^13^, was applied for simultaneous grain refinement and alloying process. A similar approach was used earlier during friction stir welding and processing where Cu^14^, and Fe^15^ powders were added during the welding and processing of Al alloys, resulting in a homogeneous distribution of intermetallic precipitates and thus enhancing mechanical properties of the processed zone. The FSA process used in the current work, utilizes the shear deformation during the solid-state processing, to distribute the additional desired alloying element into the matrix by non-equilibrium dissolution. Our previous work on non-equilibrium dissolution of Al into Al_0.1_CoCrFeNi HEA matrix^16^ and that of Cu^17^ and V^18^ into TRIP HEA matrix via FSA produced encouraging results and established the viability of this process. FSA is a more efficient way to develop new alloys, especially for HEAs, since alloy modification on the existing material can be achieved without bulk melting, formation of intermetallics and cracks. Furthermore, the microstructure after FSA (or any friction-stir-based technique) is predominantly recrystallized and hence requires no further processing (such as rolling or heat-treatment), as a result of the synergistic effects of strain, strain rate and temperature on microstructural modification. Note that FSA is a local alloying technique which cannot produce bulk materials, but upscaling of such a process is achievable by using the emerging friction-based manufacturing techniques^19^.

In the current study, we selected Fe_42.8_Mn_28.3_Cr_14.8_Ni_9.6_Si_4.5_ at.% (Ni–Si5–HEA) as the starting material and alloyed Ti using FSA. The alloying of Ti resulted in formation of Ni_3_Ti precipitates which, on one hand, strengthened the material; and, consequently, they also reduced Ni concentration around Ni_3_Ti precipitates, thus lowering the SFE enough to introduce an additional TRIP mechanism effect locally.

## Results and discussion

### Benchmark from FSP

**Figure 1:**
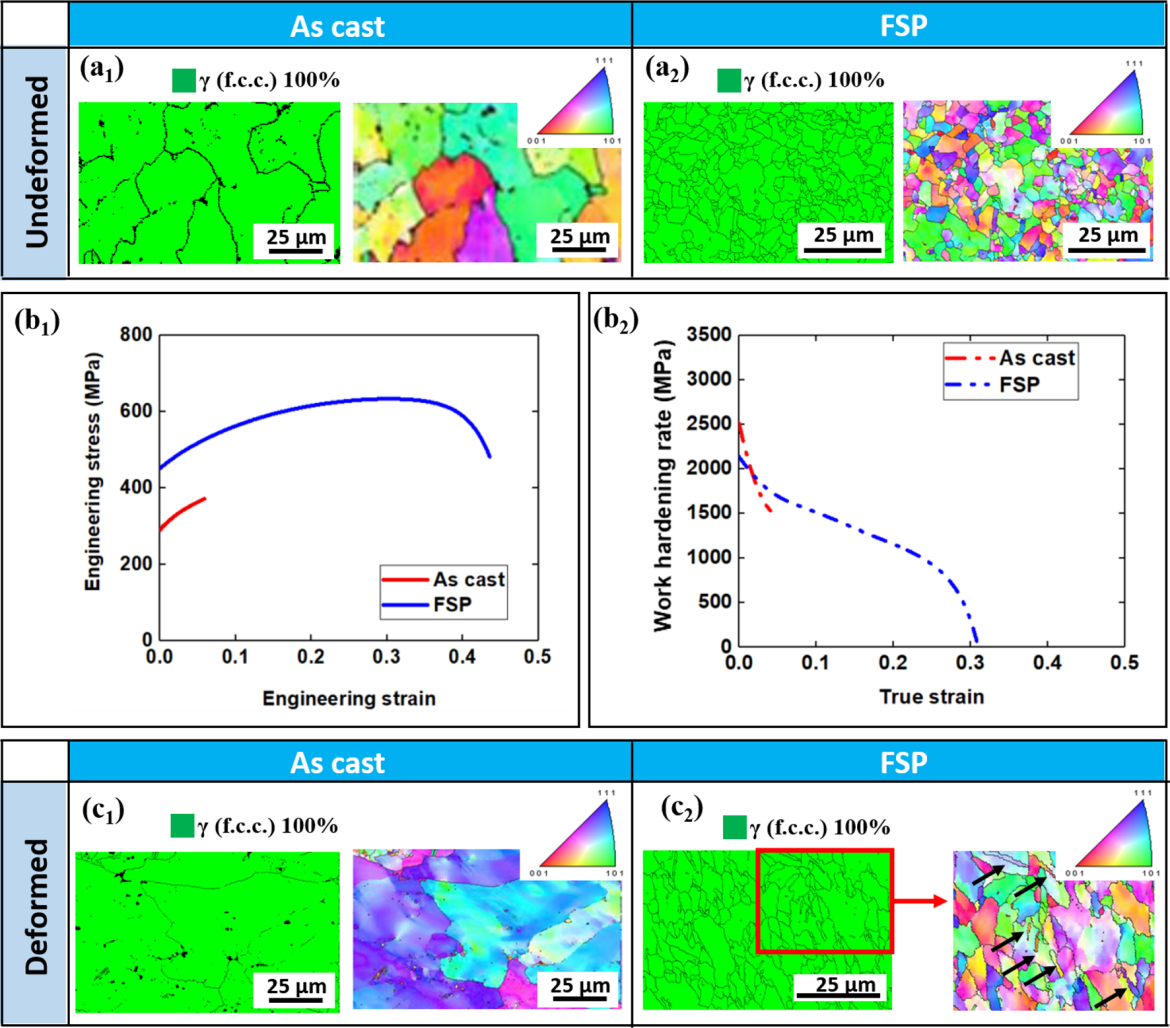
EBSD phase map and IPF map of (**a**_**1**_) as-cast and (**a**_**2**_) FSP specimen in undeformed condition; (**b**_**1**_) engineering strain–stress and (**b**_**2**_) work hardening rate curves of as-cast and FSP specimens under quasi static tensile testing load; EBSD phase map and IPF map of (**c**_**1**_) as-cast and (**c**_**2**_) FSP specimens in deformed condition. Note that deformational twins are labelled by black arrows in (**c**_**2**_).

Figure 1(a_1_–a_2_) display the microstructural variation in as-cast and FSP conditions. EBSD phase maps confirm that both as-cast and FSP conditions consist of a single phase of γ, which indicates that no phase transformation occurred during FSP. Grain refinement was observed after FSP as the average grain size of 13.7 ± 2.5 µm in as-cast condition reduced to 1.8 ± 0.6 µm in FSP condition. Mechanical behavior includes engineering stress–strain curves and work hardening rate of as-cast and FSP specimens (Fig. 1(b_1_–b_2_)). Yield strength, tensile strength and ductility increased from ~ 288 MPa, ~ 367 MPa and ~ 6% in the as-cast condition to ~ 451 MPa, ~ 645 MPa and ~ 43% in the FSP condition (Fig. 1(b_1_)). The decelerating trend in work hardening rate of FSP condition suggests that no phase transformation (TRIP) occurred during testing (Fig. 1(b_2_)). Also, there is no clear trend of work hardening rate of as-cast condition since it had limited ductility and early failure due to its coarse-grained structure (Fig. 1(b_2_)).

Figure 1(c_1_,c_2_) display the microstructural response of as-cast and FSP specimens in deformed condition. Note that the scans were taken near the fracture tip. Phase maps of both as-cast and FSP conditions after deformation confirmed that no phase transformation had occurred, thereby indicating that TRIP had not occurred. In addition, the inverse pole figure (IPF) maps of deformed FSP specimen show that deformation twins (labelled by black arrow in Fig. 1(c_2_)) formed during loading. While coarse grain size favors twinning, the lack of evidence of deformation twins in the deformed as-cast specimen might be due to the limited number of grains in the gauge section of the as-cast condition. Hence strain hardening behavior would be dependent on the orientation of the individual grains with respect to the loading axis. For the present study, limited strain seems to have been accommodated by the as-cast microstructure by the onset of TWIP before crack and fracture. Therefore, improvement in mechanical properties of the FSP condition can be attributed to grain refinement and TWIP.

### Parameter optimization for FSA

**Figure 2:**
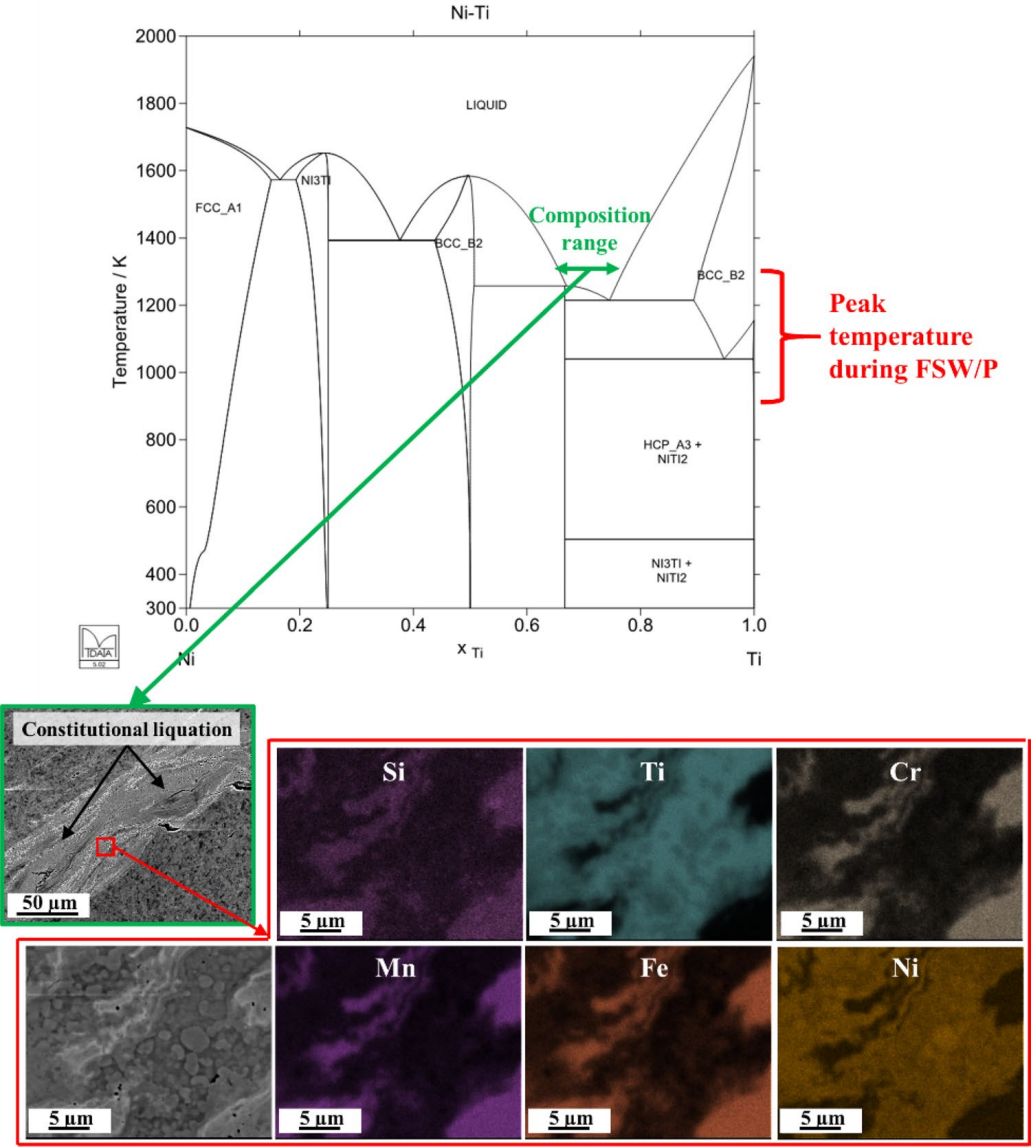
Phase diagram of Ni–Ti and SEM image and EDS map on processed region after one pass FSA displaying that constitutional liquation occurred during FSA.

We initially used a high tool rotation rate (600 RPM) to increase strain, strain rate and temperature in order to promote dissolution of Ti into the FCC matrix. However, somewhat unexpectedly, excessive heat was produced during processing. Local melting of Ti was observed in the processed region (SEM images of Fig. 2). Previous studies confirmed that peak temperature during friction stir welding/processing on steels ranged from ~ 900 to 1300 K^20–23^. We assumed that the peak temperature during FSA of Ni–Si5–HEA would also fell in the same range since Fe content is near 40 at.% in the matrix. The peak temperature during the FSA process being lower than the melting point of Ti by ~ 600–1000 K (phase diagram in Fig. 2) implies that constitutional liquation occurred when Ti and Ni mixed in a certain compositional ratio (composition range labelled in the phase diagram in Fig. 2).

Similar to observations in the present study, Sato et al.^24^ reported that constitutional liquation occurred between friction stir welding (FSW) of aluminum and magnesium alloys at excessive temperatures. Therefore, determining welding parameters that would generate lower heat during FSA is desirable. Previous researchers correlated the FSW parameters with non-dimensional peak temperature T^*^^25^ according to Eq. (1) and the constants of α and β have been reported as 0.151 and 0.097, respectively, which fit well with various aluminum alloys and steels based on the data reported in^26^. Hence, we have assumed the values of these constants for our HEA to be similar to the above-mentioned conventional alloys for this study.

1 $${T}^{*}=\alpha {log}_{10}( {Q}^{*}) + \beta $$

The non-dimensional peak temperature, T^∗^, is defined in Eq. (2)^25^. Q^*^ is the non-dimensional heat input defined in Eq. (3)^25^.

2 $${T}^{*}=\frac{{T}_{P}- {T}_{in}}{{T}_{s}- {T}_{in}}$$

3 $${Q}^{*}=\frac{{\sigma }_{8}A\omega {C}_{P}\phi }{{kU}^{2}}$$

where T_p_ is peak temperature, T_in_ is initial temperature and T_s_ is solidus temperature, σ_8_ is yield stress of the material at a temperature of 80% of solidus temperature of the material (0.8T_s_), A is the cross-sectional area of the tool shoulder, ω is tool rotational velocity, U is traverse speed, C_P_ is the specific heat capacity, ϕ is the ratio based on heat transfer between tool and workpiece, and k is thermal conductivity of the workpiece. Various parameters and coefficients need to be provided to calculate actual peak temperature, T_p_, during FSW for a given welding setup, including FSW tool dimensions, welding parameters and physical properties of the base material and tool. If welding parameters are the only variables with constant FSW tool and base material properties, the change in peak temperature can be simplified and calculated via Eq. (4), which was obtained through Eqs. (1)–(3).

4 $${T}_{P}\left(1\right)- {T}_{P}(2)=\alpha {log}_{10}\left(\frac{\upomega (1){U(2)}^{2}}{\upomega (2){U(1)}^{2}}\right)({T}_{s}-{T}_{in})$$

We assumed the solidus temperature of the alloy (T_S_) as ~ 1200 °C, and T_in_ equals 25 °C (ambient temperature). By reducing the tool rotation rate (ω) from 600 to 150 RPM along with a constant traverse speed of 50.8 mm/min, peak temperature reduced by ~ 130 °C. Combined with the fact that peak temperature during friction stir welding/processing generally ranged from 900 to 1300 K, the peak temperature during optimized FSA process (rotation rate of 150 RPM) is assumed to be lower than ~ 1200 °C (lower than constitutional liquation temperature of Ni–Ti system). Furthermore, the observation of no solidification features at 150 RPM indicated that the reduction of peak temperature by 130 °C was sufficient to avoid constitutional liquation between Ti and Ni originating from Ni–Si5–HEA matrix during FSA. Therefore, two-pass FSA with tool rotation rate of 150 RPM was conducted to dissolve Ti while avoiding constitutional liquation. EDS analysis was conducted on nugget region of double-pass FSA condition to demonstrate element distribution (see Supplementary Fig. 2). Compared with as-cast condition, elements of Cr, Mn. Fe and Ni are more homogenized, Si–Mn–Ni-rich phases were replaced with much finer Si-rich phases, and Ti was dissolved into matrix in FSA condition.

### FSA and FSA + Anneal

**Figure 3:**
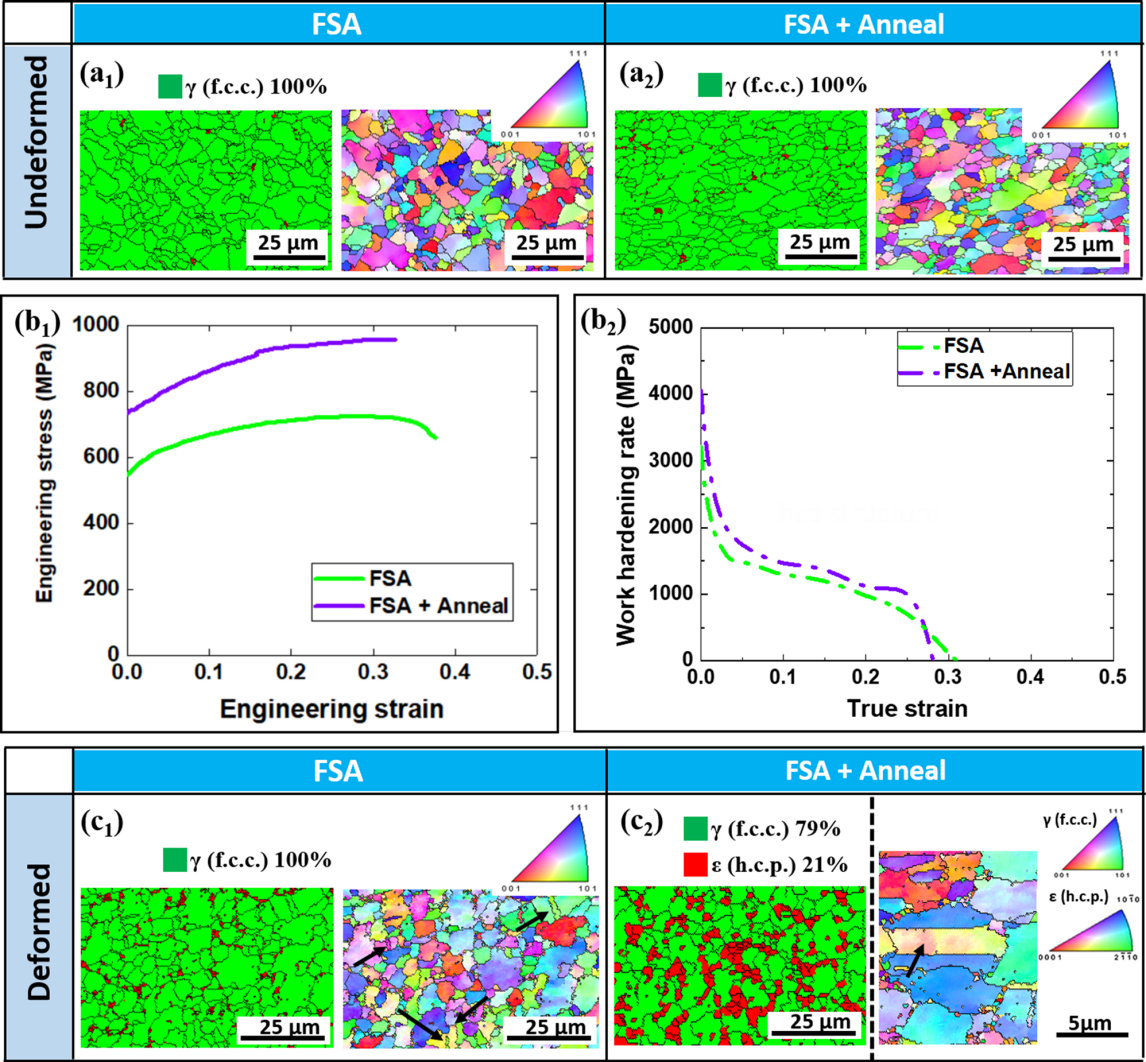
EBSD phase map and IPF map of (**a**_**1**_) FSA and (**a**_**2**_) FSA + Anneal specimens in undeformed condition; (**b**_**1**_) engineering strain–stress and (**b**_**2**_) work hardening rate curves of FSA and FSA + Anneal specimens under quasi static tensile testing load; EBSD phase map and IPF map of (**c**_**1**_) FSA and (**c**_**2**_) FSA + Anneal specimens in deformed condition. Note that deformational twins are labelled by black arrows in (**c**_**1**_ and **c**_**2**_). Also note that the red color displayed in phase map of (**a**_**1**_), (**a**_**2**_) and (**c**_**1**_) represent regions with low confidence indexes values rather than ε (HCP) phases.

**Figure 4:**
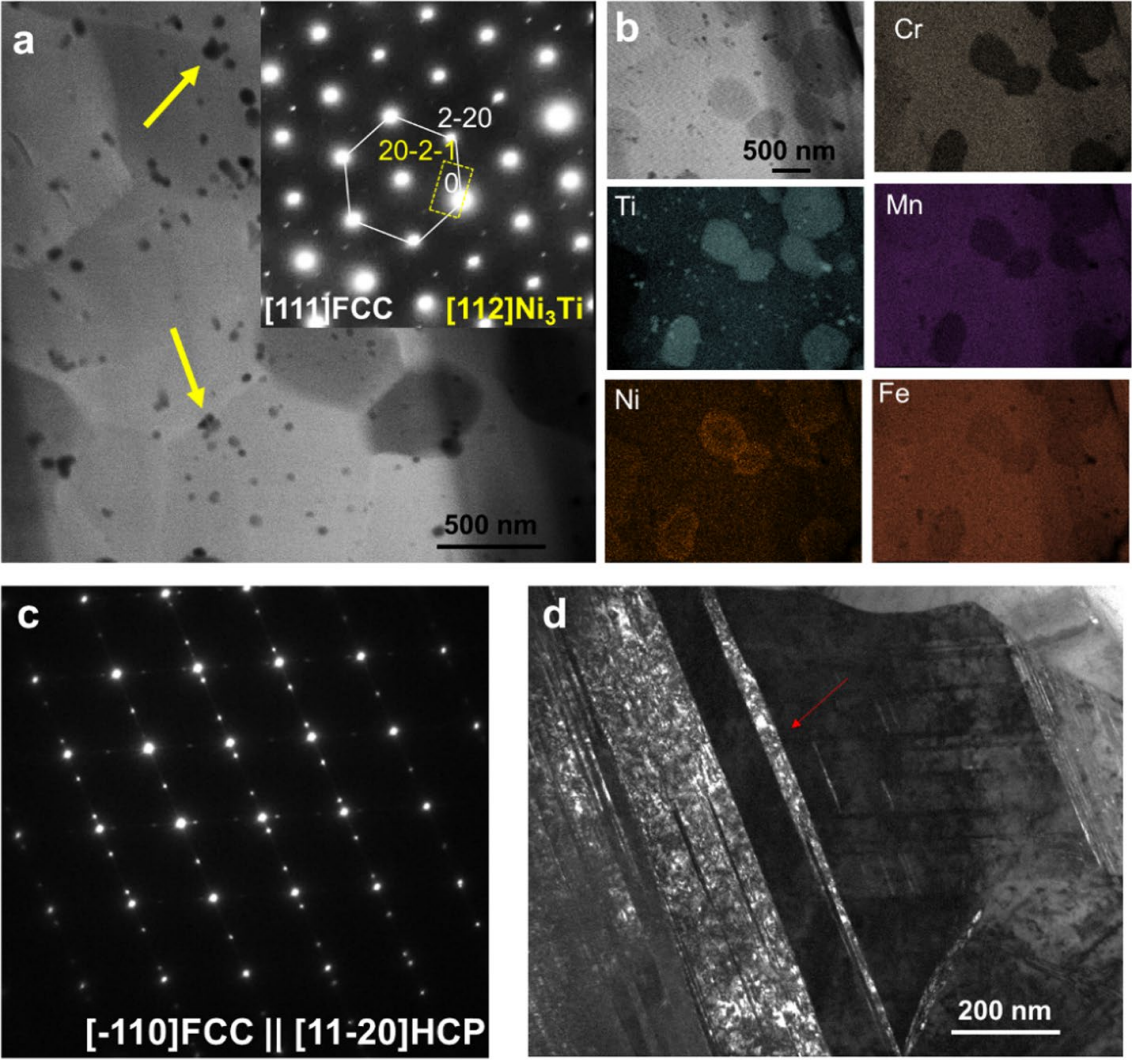
(**a**) HAADF-STEM image and corresponding SAED showing Ni_3_Ti precipitates indicated via yellow arrows after annealing and (**b**) corresponding TEM–EDS analysis revealing the presence of Ti and Ni rich precipitates. (**c**) Post-deformation SAED indicating the orientation relationship between ${<1\overline{1}0>}_{fcc}$ and ${<11\overline{2}0>}_{hcp}$ and (**d**) BF-TEM showing transformation induced nano-scale HCP phases indicated via red arrows.

FSA was conducted with optimized parameters as listed in Table 1. Then, microstructural analysis using orientation microscopy of before-and-after tensile testing was conducted on FSA specimens (Fig. 3(a_1_,c_1_)). The phase map of the deformed FSA specimen did not show any significant evidence of stress-induced phase transformation (TRIP effect); however, deformation twins (TWIP) can be observed in the IPF map of the deformed FSA specimen (Fig. 3(c_1_)). Yield strength (~ 554 MPa) and tensile strength (~ 725 MPa) of FSA specimens are both higher than the FSP condition. This is because the grain size (1.2 ± 0.5 µm) in the FSA condition with lower rotation rate and heat input was finer than that in the FSP condition. A similar trend of work hardening rate in the FSA specimen as compared to the FSP specimen from tensile testing response also suggests that TRIP was not triggered during testing of FSA specimens (Fig. 3(b_2_)). The additional annealing step was done resulting in the precipitation of Ni_3_Ti phase as confirmed in Fig. 4(a). The precipitation of this Ni enriched intermetallic phase may result in lowering of Ni concentration in the matrix surrounding these precipitates. The reduction Ni concentration can result in lowering of the stacking fault energy of the matrix and just increase the possibility of deformation by TRIP. Note that after annealing at 900 °C for 4 h, we did not observe significant grain coarsening or phase changes in the microstructure (Fig. 3(a_2_)). Yield strength and tensile strength of FSA + Anneal specimen are enhanced to ~ 736 MPa and ~ 958 MPa, respectively (Fig. 3(b_1_)). Meanwhile, work hardening rate of the FSA + Anneal specimen being more sustained as compared with those of FSA and FSP conditions (Fig. 3(b_2_)) suggests the possibility of an additional mechanism (TRIP) while deformation in the FSA + Anneal specimen during loading. Figure 3(c_2_) presents 21% of γ → ε transformation (TRIP) and deformation twins (TWIP) of the FSA + Anneal specimen via EBSD phase map and IPF map, respectively.

**Table 1:** Critical process parameters applied for FSP and FSA process.

Condition	Rotation rate (RPM)	Traverse speed (mm/min)
**FSP**		
First pass	650	50.8
Second pass	350	50.8
**FSA**		
First pass	150	50.8
Second pass	150	50.8

To further substantiate the proposed hypothesis, a detailed TEM analysis of the final condition (i.e., FSA + Anneal) specimen was conducted. The results of both pre- and post-deformation TEM analyses are presented in Fig. 4. A high annular angle dark field scanning TEM (HAADF STEM) image of the pre-deformed sample shows the fine-grained structure with dark contrast precipitates (Fig. 4(a)). In addition to a high density of extremely fine precipitates (~ 5 nm), some large second-phase grains were present. The corresponding selected area electron diffraction (SAED) pattern was recorded along [111]_*FCC*_ zone axis (Fig. 4 (a) inset). Note that the extra reflections in the SAED pattern were consistently indexed to be [112] Ni_3_Ti phase based on a D0_24_ structure. The precipitates were further characterized using EDS. Precipitates rich in Ti and Ni were clearly visible in the TEM–EDS analysis of the pre-deformed specimen (Fig. 4(b)), and hence the depletion of Ni from the matrix was achieved via annealing treatment once the matrix was saturated with Ti during FSA. The evolution of deformation structure on the tensile tested sample is presented in Fig. 4(c),(d). The SAED pattern of the deformed specimen (Fig. 4(c)) clearly demonstrates a well-recognized orientation-relationship (OR) of FCC → HCP transformation, also known as Shoji-Nishiyama OR, where ${<1\overline{1}0>}_{fcc}$ is parallel to ${<11\overline{2}0>}_{hcp}$. The corresponding dark field TEM (DF-TEM) image in Fig. 4(d) also reveals the presence of HCP phase lamellae in FCC matrix. Previous studies^27–29^ have established that in metastable FCC alloys, the formation of HCP lamellae is expected during deformation. Hence, the final microstructural condition was tuned to demonstrate the deformation-induced transformation of FCC to HCP phase.

**Figure 5:**
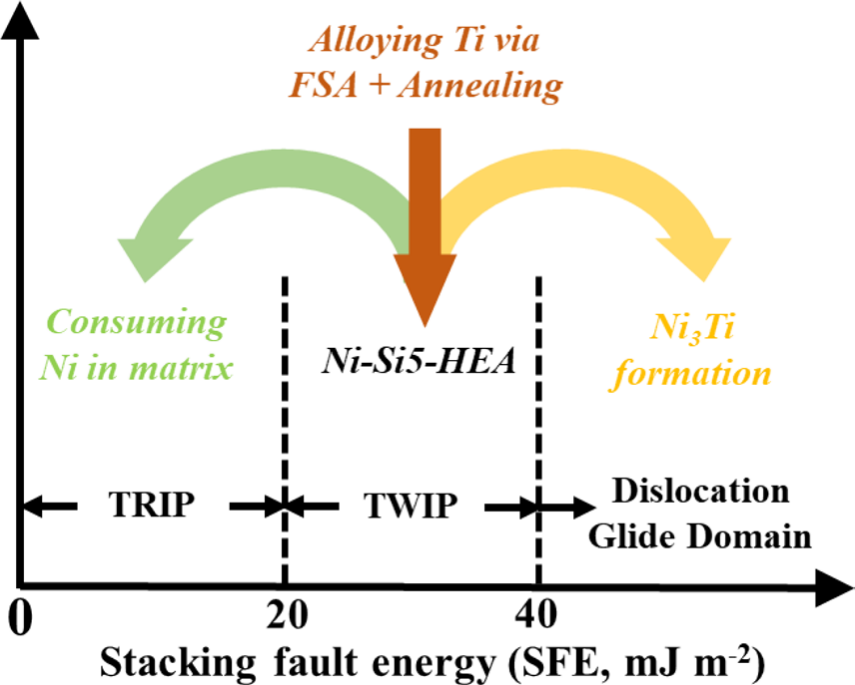
Schematic showing the proposed tuning pathway via FSA.

A possible mechanism and procedure of FSA is summarized schematically in Fig. 5. Idrissi^30^ reported that the activated plasticity mode is highly dependent on SFE values. With decreasing SFE, the observed deformation mode is well-known to be transition from dislocation glide to mechanical twinning and finally to martensitic phase transformation^31^. Generally, deformation-induced martensitic transformation is triggered when SFE < 15 mJ m^−2^^32^, mechanical twinning is triggered when 20 mJ m^−2^ < SFE < 40 mJ m^−2^, and dislocation glide becomes the predominant plastic deformation mode when SFE > 40 mJ m^−2^^33^. According to previous literature^30–33^, SFE of Ni–Si5–HEA should lie within 20–40 mJ m^−2^, based on the absense of phase transformation during straining. By introducing Ti into the system, Ni was preferentially depleted from the matrix. As a result, local reduction in SFE was expected locally below 15–20 mJ m^−2^, enabiling the onset of TRIP during tensile deformation. In addition to activation of TRIP as a strengthening mechanism, the newly-formed Ni_3_Ti precipitates also act as obstacles to dislocation glide during mechanical deformation. Meanwhile, TWIP was maintained during mechanical deformation since a certain amount of Ni was left in the remaining matrix. Local changes are expected to have led to the changes deformation mechanisms, thus we evaluated the resultant deformation behavior on work hardening behavior was observed to change.

To correlate the work hardening behavior, a strength model recently developed by Lloyd et al. was emploted to correlate work hardening behavior with expected SFE. Four parameters were developed to model the strain hardening behaviors of TRIP and TWIP steels^34^. One of the four paramters θ_0_ was shown to be related to the SFE, by the relationship shown in Eq. (5):

5 $${\theta }_{0}=17500 \times {\left(\mathrm{SFE}+5.0\right)}^{-0.6}$$

**Figure 6:**
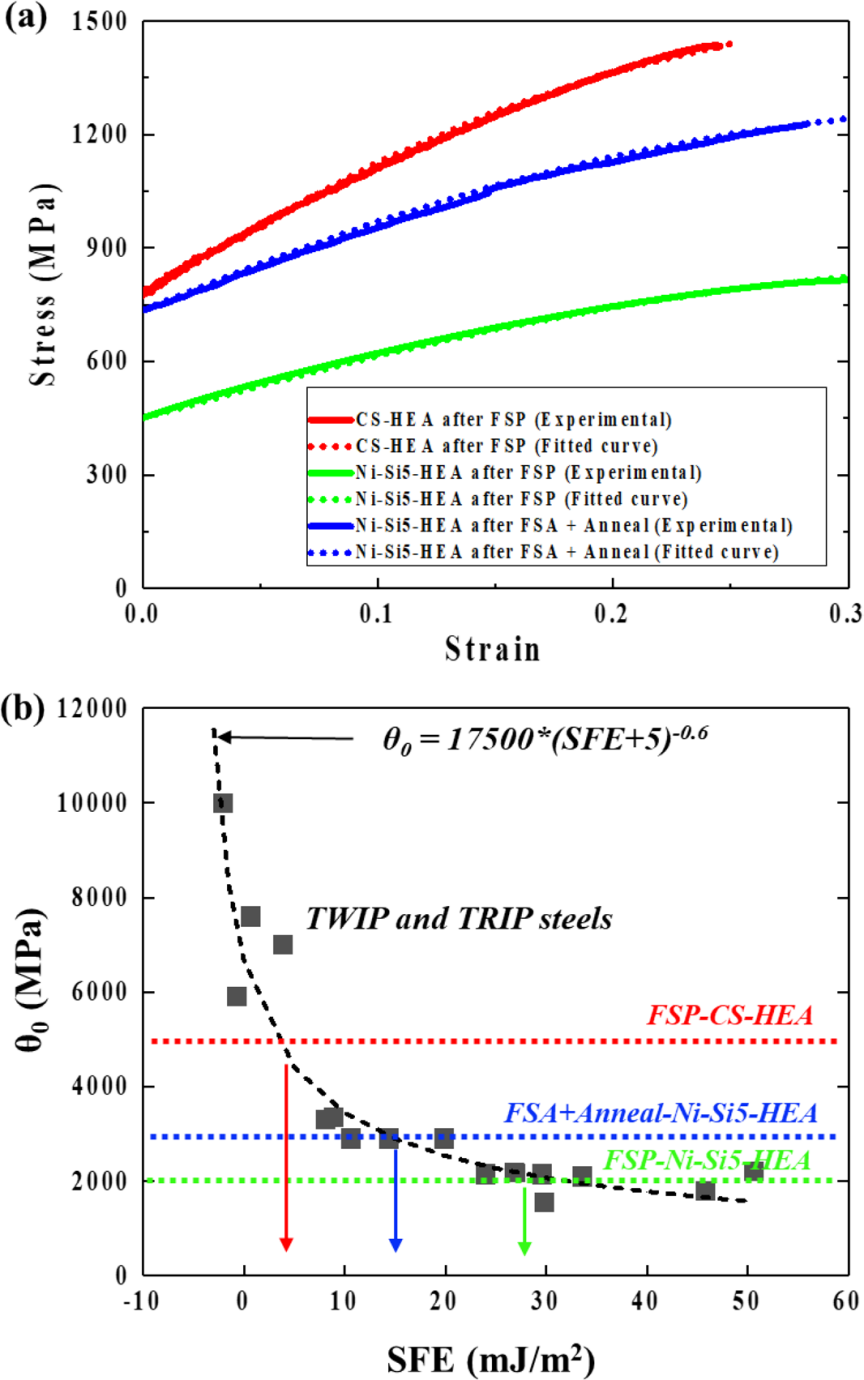
(**a**) Experimental stress–strain curves of CS–HEA and Ni–Si5–HEA in different conditions, (**b**) Correlations of θ_0_ and SFE for TRIP and TWIP steels.

Therefore, in this study, the four parameters of the model were tuned to fit the experimental stress–strain behaviors of the FSP and the FSA + Anneal conditions of Ni–Si5–HEA. By comparison, another TRIP–HEA (CS–HEA^35^) with similar chemical composition was also investigated. The experimental stress–strain curves and fitting curves are shown in Fig. 6(a). θ_0_ for CS–HEA after FSP, Ni–Si5–HEA after FSP, and Ni–Si5–HEA after FSA + Anneal is 5000, 2000 and 3000, respectively. Comparing θ_0_ of TRIP and TWIP HEAs with TWIP and TRIP steels, the estimated SFE of Ni–Si5–HEA after FSP and FSA + Anneal are shown in Fig. 6(b). Note that the estimated SFE of CS–HEA after FSP is between 0 and 10, which agrees with measured SFE of CS–HEA though neutron diffraction^35^.

## Conclusions

A novel solid-state alloying method―friction stir alloying (FSA)―was applied to introduce Ti into a TWIP HEA. The conclusions are:

- FSA parameter optimization was achieved by reducing tool rotation rate to 150 RPM Ni–Ti constitutional liquation during FSA. Subsequently, Ti was successfully dissolved into the Ni–Si5–HEA matrix during FSA.
- Annealing of the FSA specimens led to the formation of Ni_3_Ti precipitates so that precipitation hardening was introduced in this alloy system.
- Formation of Ni_3_Ti precipitates facilitated a Ni-depleted region in the vicinity, which enabled TRIP locally.
- Transformation pathway under strain was modified from stand-alone TWIP in the base material to TWIP + TRIP + PH in the FSA + Anneal specimen.
- Deformation behavior correlates well with SFE predicted by stress–strain response based on experimental observations of TWIP and TRIP.

## Materials and methods

### Materials

The starting material is as-cast Ni–Si5–HEA (Fe_42.8_Mn_28.3_Cr_14.8_Ni_9.6_Si_4.5_ at.%). The alloy was produced by vacuum arc-casting in a cold copper crucible, using pure metal powders and ingot dimensions of 300 × 100 × 6 mm^3^. The chamber was backfilled with argon to 1 atm. prior to each melt. The microstructure of as-cast Ni–Si5–HEA is detailed in Supplementary Fig. 1. As-cast Ni–Si5–HEA consists of a single γ (*FCC*) phase (Supplementary Fig. 1 (a)), but inhomogeneity in alloying elements exists. Apart from the matrix, Si–Mn–Ni-rich and Cr–Fe-lean phases (labelled by yellow circles) and Cr-rich and Ni-lean phases (labelled by green circles) were observed (Supplementary Fig. 1 (b)).

### Methods

The influence of FSP and additional alloying of Ti were differentiated by stand-alone FSP on the Ni–Si5–HEA (parameters listed in Table 1). As for the FSA process, pre-drilled holes of diameter 0.6 mm, depth 3.0 mm and gap 1.0 mm along the tool traverse direction were introduced via drilling on the as-cast Ni–Si5–HEA sheet. Ti powders of 99.9% purity and ~ 10 µm average particle size were filled into these pre-drilled holes. Note that the volume percentage of holes to processed region was ∼ 5%. According to the chemical composition of base Ni–Si5–HEA (Fe_42.8_Mn_28.3_Cr_14.8_Ni_9.6_Si_4.5_ at.%), the density of base Ni–Si5–HEA is calculated as ~ 7.3 g/cc. The density of Ti is ~ 4.5 g/cc, therefore, the volume fraction of Ti being 5% can be transferred to be mass fraction of Ti being ~ 3.1%. The nominal chemical composition of FSA (or FSA + Anneal) condition is: Fe_41.3_ Mn_27.3_ Cr_14.3_ Ni_9.3_ Si_4.3_Ti_3.5_ at.%. Afterwards, the rotating tool traversed along the pre-drilled holes filled with Ti powders. The parameters of FSA are also listed in Table 1. Differences in parameters between FSP and FSA are explained in the section *Parameter Optimization for FSA.* A W–Re based tool was used for both FSP and FSA. Pin length, pin diameter at root and tip, and shoulder diameter were 3.8 mm, 7.6 mm, 5.0 mm, and 16.0 mm, respectively. Plunge depth is 4.0 mm for both FSP and FSA process. In addition, an annealing process was conducted on FSA specimens so that Ni_3_Ti precipitates formed along with the consumption of Ni in the matrix of the FSA specimen. Annealing temperature and time were 900 °C and 4 h, respectively.

Both microstructural evolution and mechanical behavior were included in this study. Transverse cross sections of processed volume were cut by electrical discharge machining and were final-polished to 0.02 µm surface finish with colloidal silica suspension. Scanning electron microscopy (SEM), energy-dispersive X-ray spectroscopy (EDS) and electron backscattered diffraction (EBSD) analyses were carried out using FEI Nova NanoSEM 230 with EDAX Octane Elite EDS and Hikari Super EBSD detector, respectively. The SEM imaging and EDS analysis were performed at an accelerating voltage of 15 kV while EBSD analysis was performed with a step size of 0.05 μm. Lift-out samples for transmission electron microscopy (TEM) were prepared using a FEI Nova 200 dual beam focused ion beam (FIB). TEM analysis was carried out on Tecnai G2 F20 S-Twin TEM operating at 200 kV. Mini-tensile testing on as-cast material, FSP and FSA specimens was conducted at initial strain rate of 0.001 s^−1^. Width, thickness and gauge length of the specimens were 1.0, 1.0, and 5.0 mm, respectively. Mini-tensile specimens were taken from 1 mm below the surface of the processed nugget region. To maintain statistical accuracy, three specimens were tested for each condition.

## Supplementary Information

Supplementary Figures.

## Data Availability

The raw/processed data required to reproduce these findings cannot be shared at this time as the data also forms part of an ongoing study.
