# Supplementary material for: Co-introduction of precipitate hardening and TRIP in a TWIP high-entropy alloy using friction stir alloying
Source: Sci Rep. 2021 Jan 15;11:1579. doi: 10.1038/s41598-021-81350-0 (PMC7810985; doi:10.1038/s41598-021-81350-0)
Supplement: Supplementary file 1 — Supplementary Figures. [file 41598_2021_81350_MOESM1_ESM.docx]

cCo-introduction of precipitate hardening and TRIP in a TWIP high-entropy alloy using friction stir alloying

Tianhao Wang^a, b, #^, Shivakant Shukla^a, c, #^, Bharat Gwalani^a,b^, Subhasis Sinha^a,d^, Saket Thapliyal^a^, Michael Frank^a^, Rajiv S. Mishra^a,*^

^a^ Department of Materials Science and Engineering, University of North Texas, Denton, TX 76207, USA

^b^ Current address: Pacific Northwest National Laboratory, Richland, WA 99352, USA

^c^ Current address: Oak Ridge National Laboratory, Oak Ridge, TN, 37831, USA

^d^ Current address: Department of Metallurgical Engineering, IIT(BHU), Varanasi, India

*Corresponding author: email ID ― Rajiv.Mishra@unt.edu

^#^These authors contributed equally.


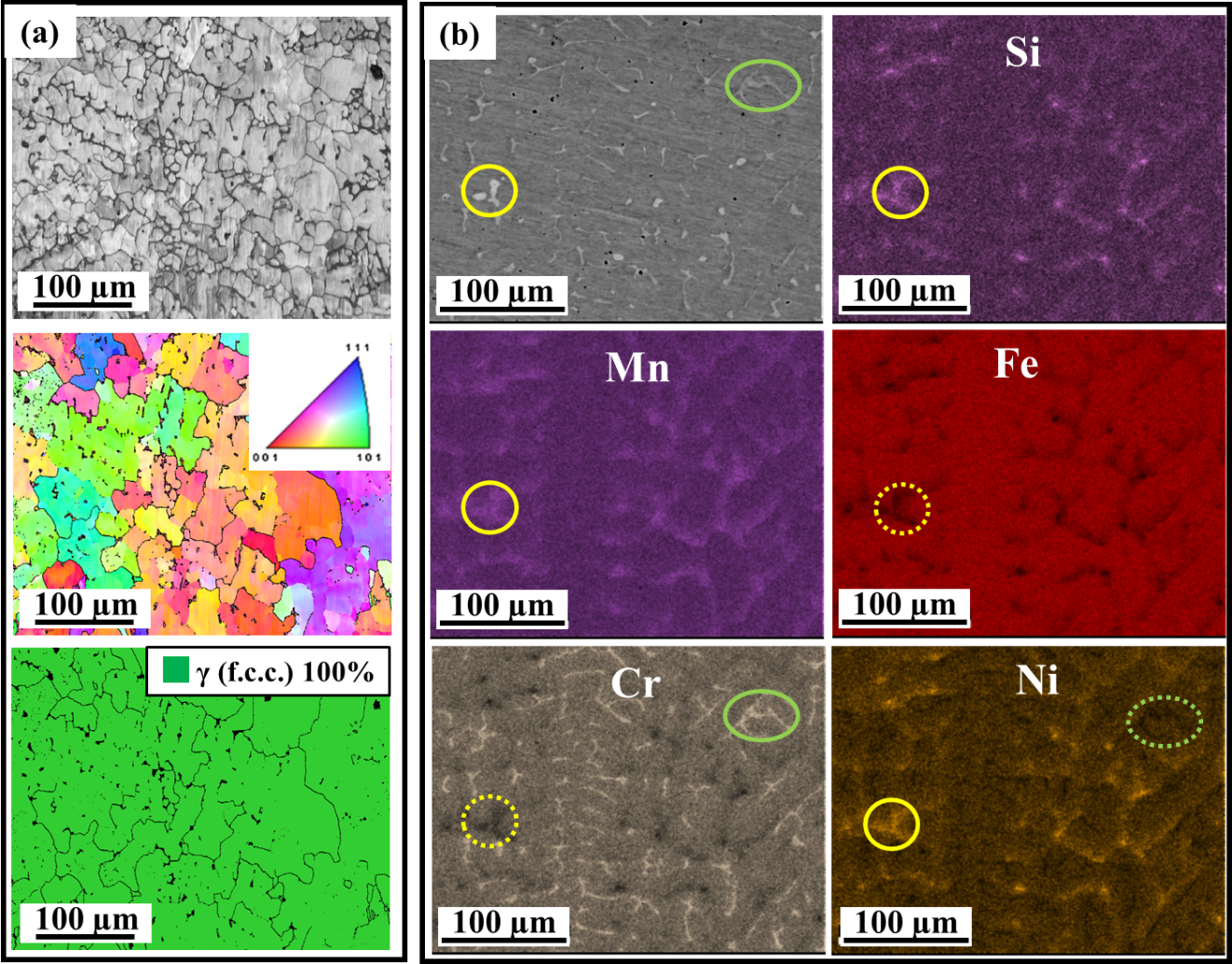


Supplementary Figure 1. (a) EBSD IQ, IPF and phase map and (b) of as-cast Ni-Si5-HEA. Note that (1) single-phase FCC, (2) alloy elements are not homogeneous; (3) Si-Mn-Ni-rich and Cr-Fe-lean phase (in yellow circles) and Cr-rich and Ni-lean phase (in green circles).


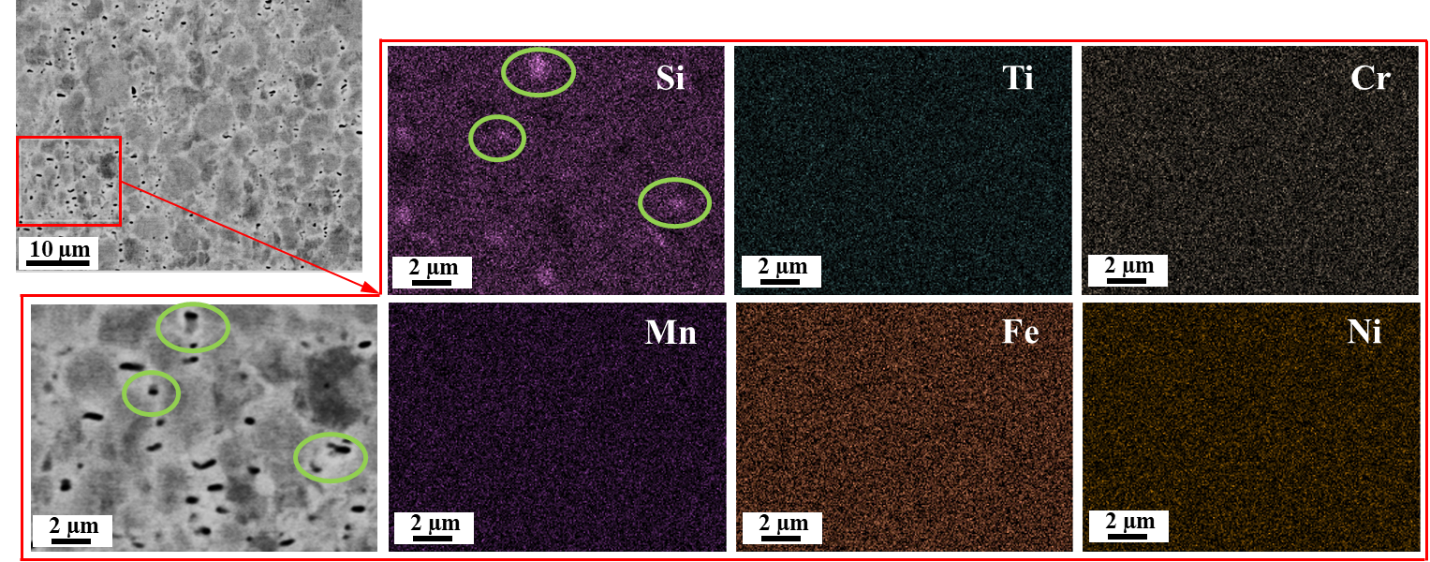


Supplementary Figure 2. EDS map of FSA condition of Ni-Si5-HEA. Note that Si-rich phase are marked (in green circles).
